# A Design Method on Durable Asphalt Pavement of Flexible Base on Anti-Rutting Performance and Its Application

**DOI:** 10.3390/ma16227122

**Published:** 2023-11-10

**Authors:** Limin Li, Enping Guo, Yuliang Lin, Zhaoyi He

**Affiliations:** 1 School of Civil and Environmental Engineering, Hunan University of Science and Engineering, Yongzhou 425199, China; li-li-min@126.com; 2 Schools of Civil Engineering, Central South University, Changsha 410075, China; 3 Schools of Civil Engineering, Chongqing Jiao Tong University, Chongqing 400074, China

**Keywords:** design method, durable asphalt pavement, flexible base, rutting and fatigue performance

## Abstract

To solve the durability of flexible base asphalt pavement, especially its anti-rutting problem, a design method on durable asphalt pavement of flexible base on anti-rutting performance was put forward in the paper, based on many experiments and calculations. Firstly, a method that asphalt could be selected according to penetration and the anti-rutting factor of its base asphalt was found, which solved the problem of the asphalt selection of the flexible base asphalt mixture design. Meanwhile, a method of skeleton-density structure gradation design was proposed based on the fractal void ratio of coarse aggregate, fractal volume of fine aggregate in coarse aggregate, penetration, fractal dimension of gradation particle size, and rutting tests, which effectively solved in advance the rutting and fatigue performance of flexible base asphalt mixtures. Then, on the basis of the fatigue damage, a calculation method of fatigue life was suggested, which solved the problem that the fatigue damage of asphalt mixtures rarely considered the combined effects of creep damage and fatigue damage. In addition, a calculation method of rutting was formulated based on vehicle dynamic load and ANSYS 16.0 software. Lastly, the feasibility of the design method on durable asphalt pavement of flexible base on anti-rutting performance was verified combining with the real engineering of a supporting project and several numerical calculations and tests.

## 1. Introduction

With the constant increase in traffic flow and axle load, the early failure of semi-rigid base asphalt pavement, such as rutting, etc., is increasingly serious in China. According to incomplete statistics [1], the occurrence rate of rutting damage is over 80% in the maintenance of high-grade highways in China. Based on the experience of existing successful applications abroad, an asphalt treated base (ATB) can solve the early failure of a semi-rigid base asphalt pavement [2,3]. However, the rutting problem is a key problem that has to be solved to use the flexible base. In fact, unreasonable pavement design is a very important reason which causes the early rut failure of asphalt pavement. Therefore, solving the rutting problem of flexible base asphalt pavement in the design stage is very necessary [4]. At present, considerable research on asphalt pavement design has been performed based on pavement performance. Huddleston et al. [5] put forward a perpetual pavement design method in 2000. The AASHTO2002 mechanical empirical design method was proposed by the National Cooperative Highway Research Program in 2004 [6]. Kamil [7] studied the performance tests and asphalt pavement design issues. Li [8] investigated the sensitivity evaluation of the mechanistic–empirical pavement design guide (MEPDG) for flexible pavement performance prediction. Zheng [9] proposed a new structure design method of durable asphalt pavement. Maadani et al. [10] investigated the environmental considerations in the AASHTO pavement design guides and declared that the current AASHTO design practice did not accurately account for the speed of passing traffic, and they resulted in a high occurrence of rutting damage, more specifically during periods of high temperature. Suresh [11] proposed flexible pavement rut prediction models from the NCAT test track structural study sections’ data. Stroup et al. [12] researched the influence of various material and traffic inputs on flexible pavement design methods for Alabama roadways, and found that the mechanistic–empirical method of flexible pavement design was strongly influenced by the hot mix asphalt thickness and the distribution of truck types within the annual average daily traffic. Yang et al. [13] studied the sensitivity of flexible pavement design to Michigan’s climatic inputs using a pavement mechanistic–empirical design, and found that climate condition, especially temperature, was an important factor that affected the performance of pavements and distress. To solve the pavement durability issues, fatigue and rutting problems are key, and various asphalt pavement design methods were performed based on specific pavement failure forms such as fatigue and rutting, etc. [14]. Much research on the fatigue and rutting problem of asphalt pavement design has been conducted [15,16,17,18,19,20,21]. At present, the gradation design methods are conducted based on practical experience and a large number of experiments, and they do not effectively solve in advance the rutting and fatigue performance of asphalt mixtures. At the same time, there is no unified standard of evaluating the rutting properties of asphalt. The calculation methods of asphalt pavement rutting have an empirical, semi-theoretical, semi-empirical, and theoretical approach. The empirical, semi-theoretical, and semi-empirical approaches require substantial observational data of pavement rutting, and they have limitations in regional and transportation conditions. Therefore, they are effectively, in practice, difficult to use. The theoretical approaches mainly focus on static analysis, and the role of vehicle dynamic loads has not been fully considered. Asphalt mixture is a typical viscoelastic material, and its fatigue damage is related to its viscoelastic properties. Currently, the evaluation of the fatigue damage of asphalt mixtures rarely considers the combined effects of creep damage and fatigue damage. Meanwhile, the asphalt pavement design method used in China was mainly developed based on a semi-rigid base on the pavement structure. Studies on the design method of durable asphalt pavement of flexible base are very limited at present [22]. Because the transportation and climate conditions in China are different from the transportation and climate conditions abroad, the existing mechanistic–empirical design methods of flexible base asphalt pavement abroad cannot be used directly in China. Hence, it is necessary to investigate systematically the design method on durable asphalt pavement of flexible base on the above problems.

## 2. Materials and Methods

In this study, Shell SBS-70 modified asphalt sourced from Maoming Guangdong province, China, and three kinds of base asphalt of Zhonghai AH-70 asphalt sourced from Zhonghai asphalt Co., Ltd., Shenzhen, Guangdong Province, China, Dagang AH-50 asphalt sourced from Dagang asphalt Co., Ltd., Tianjing, China, and Korea SK-70 asphalt sourced from Nantong Tongsha asphalt technology Co., Ltd., Nantong, Jiangsu Province, China were used, and their characteristic properties are given in Table 1 and Table 2, respectively. The coarse and fine aggregates used in the study were limestone, sourced from Shexian, Hebei Province, China. The properties of the coarse aggregates are given in Table 3. The mineral filler was crushed limestone, and its properties are shown in Table 4.

For asphalt mixtures, the rutting tests, large-scale Marshall compaction tests, freeze-thaw splitting test, small beam bending fatigue test, splitting test at 15 °C, compressive resilience modulus test at 20 °C, and mall beam bending fatigue tests were conducted according to the standard test methods for bitumen and bituminous mixtures for highway engineering (JTG E20-2011) [23] in China. The creep tests of asphalt mixtures were performed on 810MTS (Material Test System) according to the literature [25]. Dynamic creep tests of asphalt mixtures were completed using a Cooper material testing machine from Cooper Research Technology Britain in England under the action of a rectangular wave cyclic load with the frequency of 0.5 Hz according to the literature [26].

## 3. Design Methods on Flexible Base Asphalt Durable Pavement

**Figure 1:**
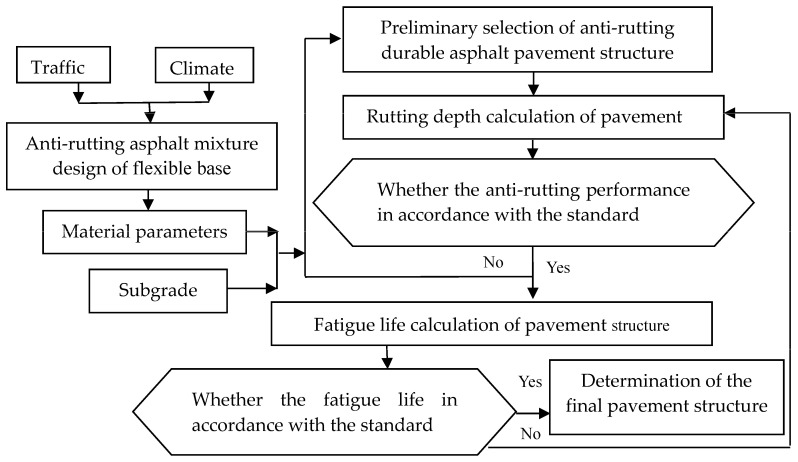
Design method on flexible base asphalt durable pavement based on rutting performance.

The design procedure of flexible base asphalt durable pavement based on rutting performance used in the paper is shown in Figure 1.

### 3.1. Anti-Rutting Asphalt Mixture Design of Flexible Base

#### 3.1.1. The Selection Method of Asphalt

The selection of asphalt is very important to the pavement performance of asphalt mixtures. According to the literature [1], the rutting performance of base asphalt mixture has good consistency with its modified asphalt mixture. For flexible base asphalt mixture, asphalt can be selected according to the high temperature performance index of the base asphalt. The base asphalt has little influence on its fatigue performance, and its penetration of asphalt has good correlation with its rutting resistance and fatigue performance. Therefore, asphalt can be selected according to penetration and the anti-rutting factor of its base asphalt.

#### 3.1.2. The Selection Method of Gradation

The skeleton-density structure of gradation has a very important role in the performance of asphalt mixtures [27]. Firstly, the flexible base gradations were selected preliminarily according to Table 5 [1]. Then, the gradations were further selected according to Table 6. The feasibility and derivation of the equations are shown in Table 6; see the literature [1].

The particle-size distributions of the fractal dimensions of coarse aggregate $D_{c}$ and the particle-size distributions of the fractal dimensions of fine aggregate $D_{f}$ can be calculated according to Equation (1).

(1) $$\left\{\begin{array}{l} p(x)=\frac{\left(x_{\min}^{3-D_{{}_{c}}}-x^{3-D_{{}_{c}}}\right)}{\left(x_{\min}^{3-D_{{}_{c}}}-x_{\max}^{3-D_{c}}\right)}\times 100\%\;x\in (x_{DCF}{}_{,}x_{\max})\\ p(x)=\frac{\left(x_{\min}^{3-D_{f}}-x^{3-D_{f}})(x_{\min}^{3-D_{c}}-x_{DCF}^{{}^{3-D_{c}}}\right)}{\left(x_{\min}^{3-D_{f}}-x_{DCF}^{3-D_{f}})(x_{\min}^{3-D_{c}}-x_{\max}^{3-D_{{}_{c}}}\right)}\times 100\%\;x\in (x_{\min\;,}\;x_{DCF}) \end{array}\right.$$

where x is the particle size, mm, p(x) is the passing rate of the particle size, %, $x_{DCF}$ is defined as the dividing size between the coarse and fine aggregates, the value of which is 4.75 mm, and $x_{\max}$ and $x_{\min}$ are the maximum and minimum particle size, respectively, mm.

Thus, according to Table 7, the dynamic stability of gradation can be obtained, and the gradations with bigger dynamic stability were selected as the target gradation. The feasibility and derivation of the equations are shown in Table 7; see the literature [1].

Based on Equation (2), on the dual logarithmic coordinate diagram, the slope λ can be obtained by using the least square method to fit the curve with the best straight line, using the results of lgp(x) and $\mathrm{lg}(x/x_{\max})$.

(2) $$p(x)={\left(x/x_{\max}\right)}^{3-D}$$

Lastly, for target gradations, the rutting tests of their asphalt mixtures were conducted using a rutting tester from China. The gradation with the maximum dynamic stability was the optimum gradation.

### 3.2. Preliminary Selection Method of Rut-Resistant Durable Asphalt Pavement

A reasonable ATB asphalt pavement can achieve the goal of durable asphalt pavement, and the asphalt pavement of ATB with a semi-rigid bottom base is the best choice of durable asphalt pavement in China [1]. For anti-rutting ATB durable asphalt pavement, the total thickness of the surface course and semi-rigid bottom base should not be less than 18 cm and 15 cm, respectively, but the semi-rigid bottom base cannot be too thick [1]. The total thickness of the surface courses and base should exceed 40 cm, and when necessary, a certain thickness of cushion should be set. In addition, the rut and fatigue property of the ATB asphalt pavement can be enhanced significantly by using high modulus asphalt concrete (AC) in the middle course.

### 3.3. Calculation Method of Rutting Depth of Pavement

**Figure 2:**
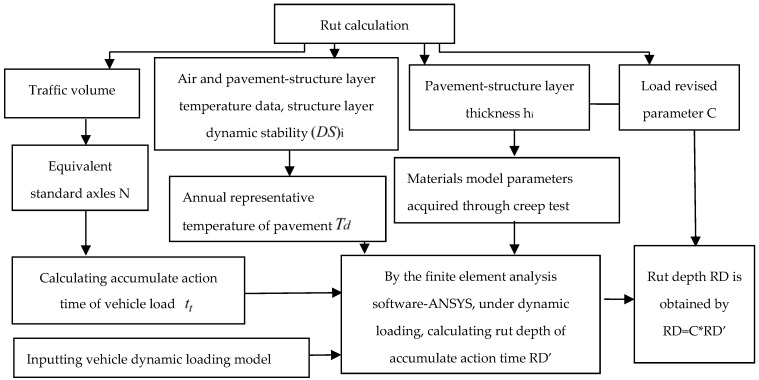
Flow charts of rut calculation method.

The design procedure of flexible base asphalt durable pavement based on rutting performance used in the paper is shown in Figure 2.

Based on previous research [1], the allowable rutting depth suggested is listed in Table 8.

#### 3.3.1. Annual Representative Pavement Temperature

The annual representative pavement temperature $T_{d}$ was obtained using Equation (3).

(3) $$T_{d}=\sum_{i=1}^{K}{\bar{T}}_{i}\frac{1}{{\left(DS\right)}_{i}}/\sum_{i=1}^{K}\frac{1}{{\left(DS\right)}_{i}}$$

where $\bar{T_{i}}$ is the monthly average pavement temperature, °C; ${\left(DS\right)}_{i}$ is the dynamic stability of the monthly average pavement temperature, times/mm. For ATB flexible base asphalt pavement, when its asphalt layer thickness is larger than 25 cm, the pavement temperature of its 10 cm asphalt surface course uses an annual representative pavement temperature, and its undersurface temperature uses an annual representative air temperature. Considering that asphalt mixture permanent deformation is very small below 20 °C [1], the annual representative air temperature is the average value of the monthly average air temperature, whose temperature value is larger than 20 °C.

#### 3.3.2. Accumulate Action Time of Vehicle Loading

The accumulate action time $t_{t}$ of vehicle loading was calculated according to Equation (4).

(4) $$t_{t}=\frac{\left[{\left(1+\gamma \right)}^{t}-1]\eta \times 0.015\times \sum_{i=1}^{k}C_{1}C_{2}n_{i}(p_{i}/p_{0}\right)}{\gamma }$$

where $n_{i}$, $p_{i}$ are the action times and its axle load, respectively; t is the design period, 15 years; $p_{0}$ is the standard axle load, 100 KN; η is the transverse load distribution factor of the lane determined by the road traffic situation; γ is the average annual growth rate of the traffic volume in the design period; and $C_{1}$, $C_{2}$ are the axle-number coefficient and wheel set coefficient, respectively.

#### 3.3.3. Finite Element Model and Load Revised Parameter C of Rutting Depth Calculation

A numerical model of subgrade and pavement was built by applying for the ANSYS software developed by American company ANSYS. ANSYS software is a large-scale universal finite element analysis software that integrates structural, fluid, electromagnetic, acoustic, and coupled field analysis. The model dimension, computational element, materials model, loading model, mesh division, and boundary conditions of the literature [1] were used in the calculation.

Asphalt pavement sustains repeated dynamic vehicle loading. In order to simplify the calculation, the repeated action times of wheel loading is replaced by accumulate action time $t_{t}$. At the same time, to reduce error, the load mode must be amended. Load revised parameter C can be obtained according to Equation (5) [1].

(5) $$C=\frac{\sum_{i=1}^{k}C^{\prime }h_{i}}{\sum_{i=1}^{k}h_{i}}$$

where $C^{\prime }$ is the ratio of the rut depth under the accumulate action time conditions to the rut depth under the repeated loading and unloading conditions under 200,000 times of axial loading, and $h_{i}$ is the thickness of each asphalt layer, m.

### 3.4. Calculation of Fatigue Life of Pavement Structure

The fatigue life of the asphalt mixture can be predicted using Equation (6) [26].

(6) $$N_{f}{}^{\prime }={\left[\pi \sigma^{2}J_{2}(\alpha_{T}\omega )\right]}^{-\beta }/(1+2\beta )$$

where *β* is a parameter related to the material aging degree, stress amplitude, loading frequency, temperature, etc. [26]; $N_{f}{}^{\prime }$ is fatigue life; σ is Cauchy stress, $\alpha_{\tau }$ is the shift factor; $J_{2}(\alpha_{T}\omega )$ is dissipation compliance; and ω is angular velocity. According to Equation (7) and Equation (8), $\alpha_{\tau }$ and $J_{2}(\alpha_{T}\omega )$ can be obtained, respectively.

(7) $$\mathrm{lg}\alpha_{T}=-\frac{C_{1}(T-T_{0})}{C_{2}+T-T_{0}}$$

(8) $$J_{2}(\alpha_{T}\omega )=\frac{1}{\eta_{2}\alpha_{T}\omega }+\frac{\eta_{2}\alpha_{T}\omega }{E_{2}^{2}+\eta_{2}^{2}{\left(\alpha_{T}\omega \right)}^{2}}$$

where $C_{1}$ and $C_{2}$ are constants; T and $T_{0}$ are the test temperature and the reference temperature, respectively, in °C; and $\eta_{2}$ and $E_{2}$ are the viscoelastic parameters of Burgers’ model. Considering the difference between the indoor fatigue test and field conditions, the fatigue life of pavement can be predicted using Equation (9) [1,26].

(9) $$N_{f}{}^{\prime }=330\times {\left[\pi \sigma^{2}J_{2}(\alpha_{T}\omega )\right]}^{-\beta }/(1+2\beta )$$

## 4. Engineering Application

### 4.1. Project Profile

**Figure 3:**
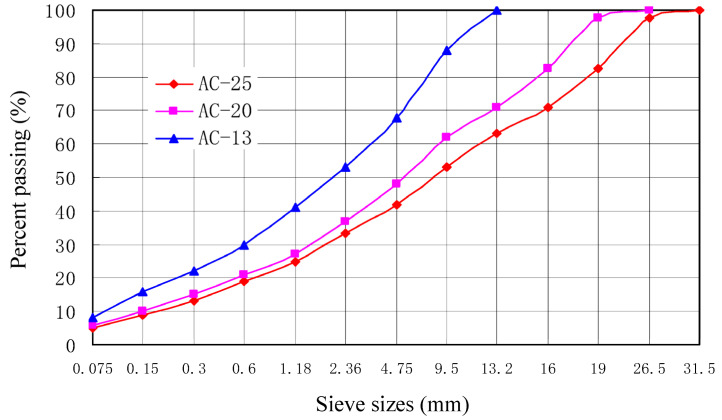
Aggregate gradations.

Vehicles of the Hanchang expressway are mainly coal transport vehicles, and its cumulative equivalent axles of design in the direction of heavy and light vehicles are 117.75 million times and 36.86 million times, respectively. The asphalt of the upper course and middle course was Shell SBS-70-modified asphalt, and the lower course used Zhonghai AH-70 asphalt. The dense-gradation asphalt mixtures of AC-13, AC-20, and AC-25 were used in the upper course, middle course, and lower course, respectively, and their aggregate gradations are shown in Figure 3. Considering the rutting performance and fatigue, the optimal oil–stone ratio of asphalt mixtures was determined comprehensively by comparing Spin Gravity Compaction and Gyratory Testing machine rotary compaction. The optimal oil–stone ratio of AC-13, AC-20, and AC-25 is 3.84%, 3.73%, and 3.41%, respectively.

**Figure 4:**
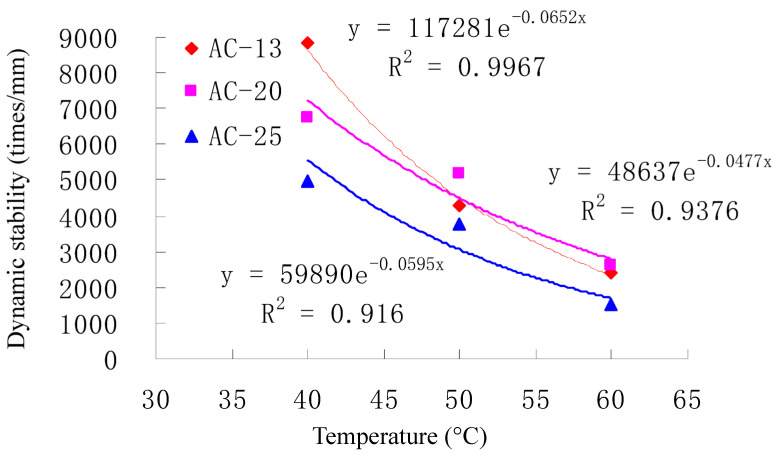
Dynamic stability of surface course asphalt mixtures.

The results of its monthly temperatures are given in Table 9. The rutting tests results of their asphalt mixtures with the optimum asphalt content are shown in Figure 4. The dynamic stability of the monthly average pavement temperature can be obtained according to the Regression equation for the dynamic stability shown in Figure 4. The annual representative pavement temperature of Hanchang highway at 40.3 °C was obtained by calculation last.

The monthly traffic volumes of the first year of the Hanchang expressway are listed in Table 10. According to Equation (4), the accumulate action time $t_{t}$ of vehicle loading is obtained, and the result is 915 million seconds.

### 4.2. Asphalt Select of Flexible Base

The penetration degree and rutting factor test results of Zhonghai AH-70 asphalt, Dagang AH-50 asphalt, and Korea SK AH-70 are listed in Table 2. Table 11 shows that the penetration degree and rutting factor values of Dagang AH-50 asphalt were the largest. Therefore, its high-temperature performance was the best. At last, Dagang AH-50 asphalt was selected based on the selection method of asphalt.

### 4.3. Determination of Anti-Rutting Gradation Design of Flexible Base

Based on the selection method of gradation, five kinds of gradation are shown in Table 11 that were selected preliminarily. The optimum asphalt content was determined using the standard Marshall method. The rutting tests of the asphalt mixtures with the optimum asphalt content were performed using a rutting tester from China. A selection of target gradation was performed, and the results are given in Table 12.

According to the selection method of gradation, based on the results of Table 12, gradation2 and gradation4 were adopted last, and their dynamic stability values were far greater than the summer hot climate area requirement (no less than 1000 times/mm) of the technical specifications for the construction of highway asphalt pavements (JTG F40-2004) [28] in China. For gradation2 and gradation4, the test results of the freeze-thaw splitting test, small beam bending fatigue test, splitting test, and compressive resilience modulus of asphalt mixtures with optimum asphalt aggregate ratio are given in Table 13.

The freeze-thaw splitting strength ratio value reflects the moisture susceptibility of the asphalt mixtures, and a higher splitting strength ratio corresponds to a higher moisture damage resistance. Intercept k of the fatigue test reflects the fatigue damage resistance of the asphalt mixtures, and a higher intercept k of the fatigue test corresponds to a higher fatigue damage resistance. The split strength, unconfined compressive strength, and compressive resilience modulus values reflect the anti-deforming property of asphalt mixtures. It can be seen from Table 13 that for two kinds of selected gradations, the moisture susceptibility of gradation2 asphalt mixtures is better than that of gradation4 asphalt mixtures, and they are able to meet the requirement of no less than 75%; the anti-fatigue performance of gradation2 asphalt mixtures is better than that of gradation4 asphalt mixtures too. According to the literature [1] and Table 13, it can be found that the split strength, unconfined compressive strength, and compressive resilience modulus values of the selected gradation asphalt mixtures were higher than that of the semi-rigid base asphalt mixture. The split modulus values of the selected gradation asphalt mixtures were far lower than that of the semi-rigid base asphalt mixture. Therefore, their asphalt mixtures have an excellent anti-deforming capability. Considering its rutting performance and fatigue integration, gradation2 was selected last.

### 4.4. Determination of Asphalt Structure Flexible Base Durable Asphalt Pavement

#### 4.4.1. Rutting Depth Calculation of Flexible Base Durable Asphalt Pavement 

**Figure 5:**
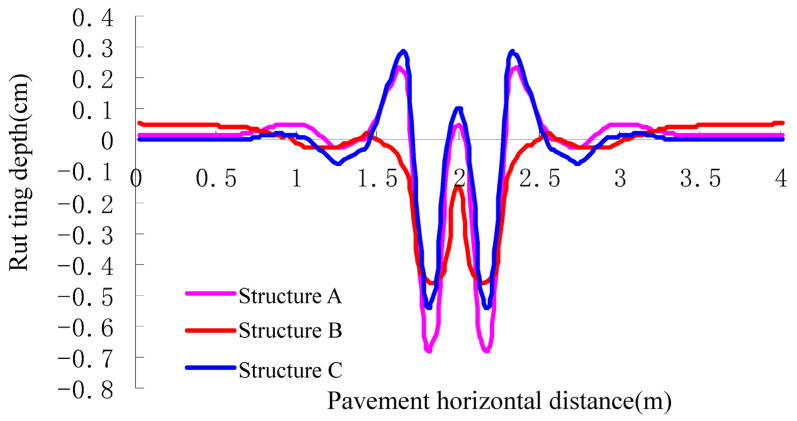
The rutting depth calculation results of asphalt pavement of flexible base.

Based on the preliminary selection method of rut-resistant durable asphalt pavement, three kinds of pavement structures shown in Table 14 were selected, and their material parameters are listed in Table 15. The friction angle of the lime soil subgrade is 22° and 16°, respectively, and its cohesive force is 55 kPa and 30 kPa, respectively [1]. According to the calculation method of the rutting depth of pavement, a numerical model of subgrade and pavement was built using ANSYS software. The results of the creep tests of the asphalt mixtures with an optimum oil–stone ratio at different temperatures are given in Table 16. The results of rut depth structure A, structure B, and structure C were obtained as shown in Figure 5.

The results show that the rutting depths of structures A, B, and C are 9.2 mm, 5.2 mm, and 8.1 mm, respectively, and they can all meet the rutting depth of the pavement design shown in Table 8. At the same time, structure B has the best anti-rutting performance.

#### 4.4.2. Fatigue Life Calculation of Flexible Base Durable Asphalt Pavement

The equivalent fatigue temperature T can be obtained according to Equation (10) [1].

(10) $$T=\sum_{i=1}^{12}T_{i}D_{i}/D$$

(11) $$T_{i}=6.848+0.7503T_{a}+0.0091T_{a}H-0.1024H+0.00324H^{2}-0.0001547H^{3}$$

(12) $$N_{f}=330k\sigma^{-b}$$

(13) $$D_{{}_{i}}=1-Ae^{BN_{i}/N_{f}}$$

(14) $$D=\sum_{i=1}^{12}D_{i}$$

where $D_{i}$ and D are yearly fatigue damage and monthly fatigue damage, respectively; $T_{i}$ and $T_{a}$ are the monthly mean pavement temperature and air temperature, respectively; H is the depth from the road surface; $N_{i}$ and $N_{f}$ are the number of loading repetitions and fatigue life under stress σ of the asphalt mixture, respectively; A and B are the regression coefficients of the residual stiffness modulus $S_{N}{}_{i}/S_{0}$ and recycle ratio $N_{i}/N_{f}$ of the small beam bending fatigue test of the asphalt mixture, respectively; and k and b are constants.

**Figure 6:**
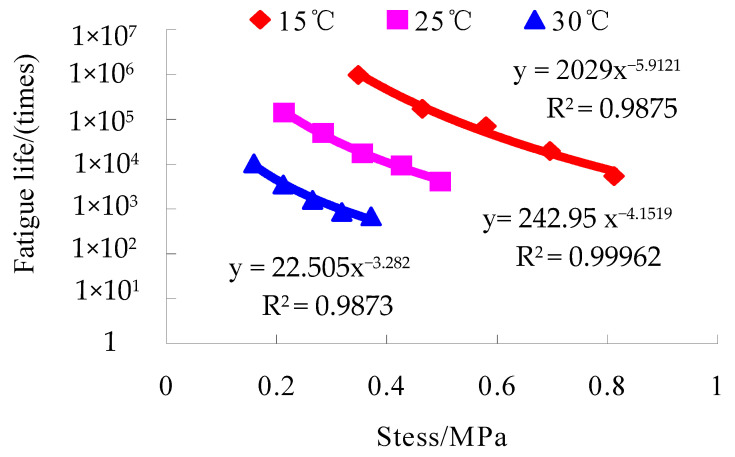
The relationship between stress and fatigue life at different temperatures.

**Figure 7:**
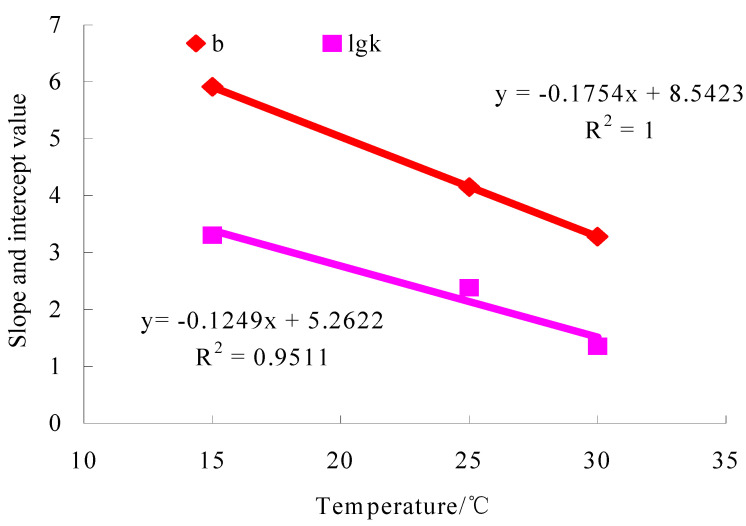
The influence of temperature on the slope and intercept of $\mathrm{lg}N_{f}$~lgσ straight lines.

**Figure 8:**
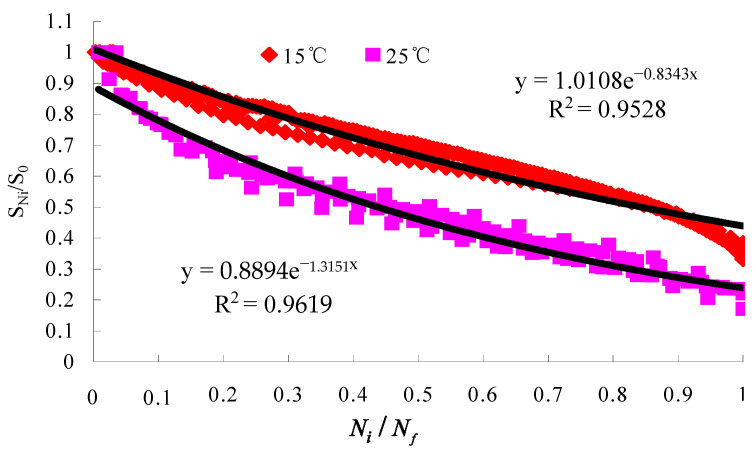
The relationship between cyclic ratio and residual modulus at different temperatures.

The test results of the small beam bending fatigue tests of the lower course asphalt mixtures with the optimal oil–stone ratio are shown in Figure 6, Figure 7 and Figure 8.

The constants k and b were obtained according to Figure 7, and $T_{i}$ was obtained according to Equation (11). A,B were obtained using the interpolation method based on Figure 8. Asphalt bottom stress σ was calculated by using Shell design software BISAR3.0, and the pavement structure analysis model of the literature [1] was used in the calculation. $N_{f}$,$D_{i}$ were calculated based on Equation (12) and Equation (13), respectively. Their calculation results were given in Table 17. At last, the equivalent fatigue temperatures of structures A, B, and C were obtained according to Equation (10) and Equation (14), respectively, and the results were 35.2 °C, 35.1 °C, and 35 °C, respectively.

**Figure 9:**
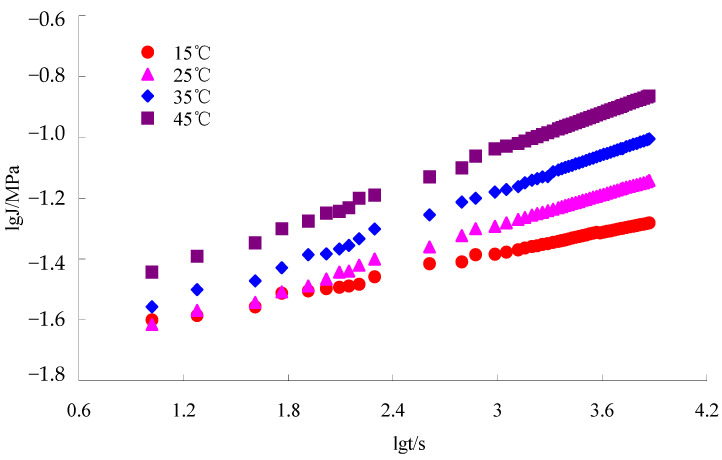
Double logarithmic curve of asphalt mixture between creep compliance and load time.

The test results of the dynamic creep tests of the lower course asphalt mixtures with the optimal oil–stone ratio are shown in Figure 9.

Based on the reference temperature *T*_0_ = 25 °C, the $\mathrm{lg}\alpha_{T}$ of different temperatures can be obtained using the horizontal shift function in Origin 9.0 software. According to Equation (4), $C_{1}$ and $C_{2}$ were obtained using the linear fitting function in 1stopt 15.0 software. Then, $\alpha_{T}$ at a different temperature was calculated using Equation (7). According to Equation (8) and Table 14, the dissipation compliance $J_{2}(\alpha_{T}\omega )$ of structures A, B, and C were obtained, and their results were 2.55 × 10^−5^, 2.51 × 10^−5^, and 2.6 × 10^−5^, respectively. Based on Equation (15) and Figure 6, the result of β=0.64 was obtained using the linear fitting function in 1stopt software. For equivalent fatigue temperature, the asphalt bottom stress of structures A, B, and C were calculated using Shell design software BISAR3.0, and the results are 0.654 × 10^−5^ MPa, 0.591 × 10^−5^ MPa, and 0.611 × 10^−5^ MPa, respectively. At last, according to Equation (9), the fatigue lives of structures A, B, and C were obtained, and their results are 1.68 × 10^11^ times, 2.1 × 10^11^ times, and 1.89 × 10^11^ times, respectively. Their fatigue lives are all far bigger than the design load, and structure B has the best anti-fatigue performance.

(15) $$N_{f}={\left[\pi \sigma^{2}J_{2}(\alpha_{T}\omega )\right]}^{-\beta }/(1+2\beta )$$

At last, considering the lack of rock asphalt-modified asphalt, the structure C was selected as an asphalt structure flexible base durable asphalt pavement.

### 4.5. Results and Discussion

**Figure 10:**
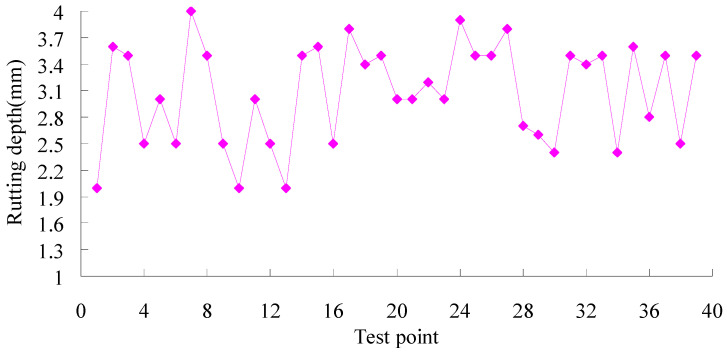
Rutting depth test results of pavement.

The tracking tests of rutting depth were conducted according to the Field Test Methods of Highway Subgrade and Pavement (JTG 3450-2019) [29] in China after the Hanchang expressway was open to traffic for two years, and the results are shown in Figure 10.

It can be seen from Figure 10 that the rut depths of pavement are all less than 4 mm, which indicates that the pavement has good anti-rutting ability. At the same time, early failures such as cracks, looseness, pits, subsidence, oil leakage, etc., did not occur after the Hanchang expressway was open to traffic for two years. The results indicate that the pavement has excellent road performance.

The tracking tests of the Hanchang expressway were conducted over only two years, and the application effect of the design method on durable asphalt pavement of flexible base on anti-rutting performance needs to be further verified.

## 5. Conclusions

(1) Considering that the skeleton-density structure could enhance the rutting and fatigue performance of the ATB asphalt mixture, a recommended method for designing the gradation of the ATB asphalt mixture was put forward, based on a fractal void ratio of the coarse aggregate $V_{C0}$, fractal volume of the fine aggregate in the coarse aggregate $V_{f}$, penetration of asphalt ZRD, fractal dimension of the gradation particle size D, and rutting test. The performance test results of their asphalt mixtures indicated that the gradation design method can solve the performance balance, such as rutting and fatigue, etc., of ATB asphalt mixtures.
(2) Based on the coupling action of vehicle dynamic loading and the pavement, the methods for calculating the representative temperatures, the time-series of the vehicle loading, and loading revised parameters, a calculation method for rutting prediction is formulated based on ANSYS software. The best pavement structure of resisting rutting can be decided by using the calculation method of rutting prediction. The rutting problem can be solved by using this method when the pavement is designed.
(3) The calculation method for fatigue life prediction is put forward based on fatigue damage. It can reflect the asphalt mixture nature of viscoelstic fatigue damage, and it overcomes the defects of typical elstic fatigue damage. At the same time, it can consider the pavement fatigue property comprehensive influences that are caused by climate, traffic, and pavement structure.
(4) The engineering application of the design method on durable asphalt pavement of flexible base on anti-rutting performance was described in detail, and it can realize the integration of the materials and structures of rutting and fatigue control during the design phase. The test results indicate that it is reasonable and practical. It is expected that more engineering project verifications will be conducted in future studies.

## Figures and Tables

**Table 1:** Properties of Shell SBS-70 modified asphalt.

Properties		Criteria	Test Value	Methods
Ductility at 5 °C (cm)		≥30	51	T0604-2011 [23]
Penetration at 25 °C (0.1 mm)		60–80	77	T0605-2011 [23]
Penetration index		−0.6–0.2	−0.18	T0604-2011 [23]
Rutting factor G/sinδ (kPa)		≥1.0	1.45	AASHTOT315
Softening point (°C)		≥48	51	T0606-2011 [23]
After the thin film oven test (TFOT) (163 °C, 5 h)	Mass loss (%)	<1.0	0.04	T0609-2011 [23]
	Ductility at 5 °C (cm)	≥30	43	T0604-2011 [23]
	Penetration ratio at 25 °C (%)	≥55	84.4	T0605-2011 [23]

**Table 2:** Properties of base asphalt.

Properties		Zhonghai AH-70	Korea SK-70	Criteria of AH-70, SK-70	Dagang AH-50	Criteria of AH-50	Methods
Ductility at 10 °C (cm)		23.5	24.7	≥20	18.2	≥15	T0604-2011 [23]
Rutting factor G/sinδ (kPa)		1.38	1.03	≥1.0	2.18	≥1.0	AASHTOT315
Penetration degree at 25 °C (0.1 mm)		68	72	60–80	56	40–60	T0605-2011 [23]
Penetration index		−0.9	−0.82	−1.5–1.0	0.81	−1.5–1.0	T0604-2011 [23]
Softening point (°C)		50	52.2	≥47	59.3	≥49	T0606-2011 [23]
After the thin film oven test (TFOT) (163 °C, 5 h)	Mass loss (%)	−0.18	−0.16	±0.8	−0.29	±0.8	T0609-2011 [23]
	Ductility at 10 °C (cm)	8.0	8.7	≥6	6.6	≥4	T0604-2011 [23]
	Penetration degree ratio at 25 °C (%)	≥62.5	64.5	≥58	65.2	≥60	T0605-2011 [23]

**Table 3:** Coarse aggregate properties.

Technical Indexes	Results	Criteria	Methods
Crush value (%)	20.0	≤26	T0316-2005 [24]
Losses of the Los Angeles Abrasion Test (%)	19.5	≤28	T0317-2005 [24]
Impact value (%)	17	≤30	T0322-2000 [24]
Mud content (%)	0.6	≤1	T0310-2005 [24]
Asphalt adhesion (graduation)	4	≥4	T0616-1993 [24]
Water absorption (%)	0.36	≤2	T0307-2005 [24]
Firmness (%)	4.5	≤12	T0314-2000 [24]

**Table 4:** Mineral filler properties.

Properties	Apparent Density (t/m^3^)	Water Content (%)	Hydrophilic Coefficient	Size Distributions (%)		
				<0.075 mm	<0.15 mm	<0.6 mm
Results	2.715	0.4	0.85	71.1	73	100
Criteria	≥2.50	≤1	<1	75–100	90–100	100
Methods	T0352-2000 [24]	T0350-1994 [24]	T0353-2000 [24]	T0351-2000 [24]		

**Table 5:** Selection range of flexible base gradation.

Sieve Sizes (mm)		0.075	0.15	0.3	0.6	1.18	2.36	4.75	9.5	13.2	16	19	26.5	31.5	37.5
ATB25	Upper limit	7	10	12	17	21	29	39	53	62	69	76	96	100	100
	Lower limit	3	4	6	10	13	20	26	38	47	52	64	91	100	100
ATB30	Upper limit	7	10	14	17	22	29	39	51	59	66	70	85	96	100
	Lower limit	2.5	4	7	9	13	19	25	34	44	49	58	75	91	100

**Table 6:** The standard of criterions of skeleton-density structure for flexible base gradation.

Gradation Type	Fractal Void Ratio of Coarse Aggregate *V_C_*_0_	Fractal Volume of Fine Aggregate in Coarse Aggregate *V_f_*	Criteria
ATB25	$V_{C0}=0.0911(D_{f}-D_{c})+0.0775$	$V_{f}=-0.2983(D_{f}-D_{c})+0.1322$	$V_{C0}>V_{f}$
ATB30	$V_{C0}=-0.0187(D_{f}-D_{c})+0.0965$	$V_{f}=-0.2626(D_{f}-D_{c})+0.112$	

**Table 7:** The calculation of dynamic stability of flexible base gradation.

Gradation Type	Dynamic Stability *DS* (mm/times)	Penetration of Asphalt *ZRD*	Fractal Dimension of Gradation Particle Size *D*
ATB25	$DS=-274573+226275D-45635D^{2}-19ZRD-0.63ZRD^{2}$	Test results according to T0605-2011 [23]	D=3−λ
ATB30	$DS=-445017+430256D-87017D^{2}-2597ZRD+19.8ZRD^{2}$		

**Table 8:** Allowable rutting depth of pavement design.

Road Grades	Expressway	Other High-Grade Roads	
		Non-Crossing Section	Intersection Section
Rutting depth (mm)	10–15	15–20	25–30

**Table 9:** Results of monthly temperatures.

Month	1	2	3	4	5	6	7	8	9	10	11	12
Maximum air temperature (°C)	10.5	13.5	21.4	33.4	34.2	41.5	39.8	36.8	38.6	30.2	24.2	15.2
Minimum air temperature (°C)	−9.2	−4.2	5.3	15.4	25.7	32.2	30.7	25.3	26.1	19.5	6.8	−7.6
Average air temperature (°C)	5.4	8.2	14.6	25.8	30.4	33.4	37.2	32.7	31.9	23.4	15.3	7.7

**Table 10:** Annual traffic volume statistics of the first year.

Load	1.5T	2T	2.5T	4T	5T	8T	10T	11.5T	14.5T	15T	20T	>20T
January	9122	988	795	1099	897	1725	767	777	912	4321	6925	8227
February	9295	1000	714	1142	953	2225	759	835	936	4831	6763	7730
March	16,299	1556	1112	1779	1808	4218	1639	1802	2021	7513	10,518	12,020
April	20,370	1784	1275	2039	1728	4032	1525	1677	1881	6587	9221	10,539
May	19,281	1831	1308	2092	1588	3704	1452	1597	1791	7417	10,383	11,867
June	18,095	1701	1215	1944	1425	3325	1365	1502	1684	7608	10,651	12,173
July	18,570	3327	2377	3802	1160	2706	1603	1764	1977	10,905	15,267	17,448
August	20,791	2004	1432	2290	1071	2499	1850	2035	2282	14,215	19,901	22,744
September	21,535	3663	2616	4186	3594	8385	1793	1973	2212	13,133	18,386	21,012
October	17,035	2176	1554	2487	1562	3644	1428	1571	1761	12,043	16,860	19,268
November	21,163	2833	2024	3238	2332	5442	1822	2004	2247	13,674	19,143	21,878
December	20,053	3495	2496	3994	2377	5546	1698	1868	2095	12,019	16,826	19,230

**Table 11:** Selected preliminarily gradation.

Sieve Sizes (mm)	0.075	0.15	0.3	0.6	1.18	2.36	4.75	9.5	13.2	16	19	26.5	31.5	37.5
Gradation1	4.5	6	8.5	13	17	23.5	30	41	51	57.5	66	95	100	100
Gradation2	4	6.5	9.5	13	17.5	23.5	30	41	52	58	70	95	100	100
Gradation3	4	5	7	12	14.5	23	29.5	46	59	65	73	92	100	100
Gradation4	4	6.5	9.5	13	17.5	23.5	30	41	49.5	55	62.5	80	95	100
Gradation5	3	5	7	10	14	21	29	43	53	61	67	84	93	100

**Table 12:** Test and calculation results of optimized gradation.

Gradation		1	2	3	4	5
Fractal void ratio of coarse aggregate *V_C_*_0_		0.098	0.1045	0.0879	0.0936	0.0953
Fractal volume of fine aggregate in coarse aggregate *V_f_*		0.0725	0.0723	0.0821	0.0798	0.0898
*V_C_*_0_ − *V_f_*		0.0255	0.0322	0.0058	0.0138	0.0055
Particle-size distributions fractal dimensions of fine aggregate *D_f_*		2.5284	2.5226	2.4943	2.5225	2.4634
Particle-size distributions fractal dimensions of coarse aggregate *D_c_*		2.3352	2.3298	2.3476	2.3903	2.3852
*D_f_* − *D_c_*		0.1932	0.1928	0.1467	0.1322	0.0782
Penetration at 25 °C (0.1 mm) *ZRD*		56	56	56	56	56
Fractal dimension of gradation particle size *D*		2.5034	2.5023	2.4579	2.5108	2.4412
Dynamic stability (times/mm)	Calculation results	2849	2851	2855	3365	3410
	Test results	2810	2855	2796	3440	3385

**Table 13:** Test results of performance tests of asphalt mixtures.

Gradation	Freeze-Thaw Splitting Strength Ratio (%)	Intercept k of Fatigue Test	Split Strength (MPa)	Split Modulus (MPa)	Unconfined Compressive Strength (MPa)	Compressive Resilience Modulus (MPa)
Gradation2	88.1	1.8464	1.179	155.45	6.17	1715
Gradation4	87.5	1.6858	1.328	168.91	7.03	1725

**Table 14:** Rut-resistant durable asphalt pavement structure.

Structure A	Structure B	Structure C
4 cm Shell SBS-70-modified asphalt mixtures of AC-13	4 cm Shell SBS-70-modified asphalt mixtures of AC-13	4 cm Shell SBS-70-modified asphalt mixtures of AC-13
6 cm Shell SBS-70-modified asphalt mixtures of AC-20C	6 cm rock asphalt-modified asphalt mixtures of AC-20	6 cm Shell SBS-70-modified asphalt mixtures of AC-20C
8 cm Zhonghai AH-70 asphalt mixtures of AC-25	8 cm Zhonghai AH-70 asphalt mixtures of AC-25	8 cm Zhonghai AH-70 asphalt mixtures of AC-25
8 cm Dagang AH-50 asphalt mixtures of ATB-25	8 cm Dagang AH-50 asphalt mixtures of ATB-25	8 cm Dagang AH-50 asphalt mixtures of ATB-25
20 cm Cement Stabilized aggregate	20 cm Cement Stabilized aggregate	20 cm Cement Stabilized aggregate
20 cm Lime soil	20 cm Lime soil	20 cm lime-flyash stabilized aggregate
subgrade	subgrade	20 cm Lime soil
		subgrade

**Table 15:** Material parameters.

Materials	Elastic Modulus (Mpa)	Poisson Ratio	Density (kg/m^3^)
Lime soil	550	0.3	1930
subgrade	48	0.4	1900
Shell SBS-70-modified asphalt mixtures of AC-13	1400	0.25	2600
Shell SBS-70-modified asphalt mixtures of AC-20C	1200	0.25	2500
Rock asphalt-modified asphalt mixtures of AC-20	2500	0.25	2500
Zhonghai AH-70 asphalt mixtures of AC-25AC-25C	1000	0.25	2500
Dagang AH-50 asphalt mixtures of ATB-25	1200	0.25	2500
Cement Stabilized aggregate	1500	0.2	2400
Lime-flyash stabilized aggregate	1400	0.25	2000

**Table 16:** Parameters of Burgers’ model with optimum oil–stone ratio at different temperatures.

Asphalt Mixtures Type	Temperature (°C)	*E*_1_ (kg/cm^2^)	*E*_2_ (kg/cm^2^)	*η*_1_ (kg/cm^2^·s)	*η*_2_ (kg/cm^2^·s)
Shell SBS-70-modified asphalt mixtures of AC-13	60	1000	450	34,652	1,684,652
	50	1200	612	54,982	1,559,747
	35	3000	597	111,357	1,641,532
	20	4000	1365	74,856	3,270,865
Shell SBS-70-modified asphalt mixtures of AC-20	60	800	511	9761	1,412,325
	50	1450	707	29,345	1,333,541
	35	2200	461	99,462	1,806,501
	20	3500	1647	50,317	2,334,658
Rock asphalt-modified asphalt mixtures of AC-20	60	1220	767	11,783	1,664,630
	50	2370	837	33,179	1,424,289
	35	3320	659	145,858	1,986,599
	20	6550	1992	62,278	3,130,207
Zhonghai AH-70 asphalt mixtures of AC-25	60	600	494	8890	1,532,292
	50	1200	821	11,006	1,297,562
	35	2050	1322	9057	2,003,491
	20	2800	1597	16,652	2,628,311
Dagang AH-50 asphalt mixtures of ATB-25	60	580	487	8245	149,813
	50	1180	770	14,372	1,256,866
	35	1860	1087	72,784	1,819,868
	20	2780	1394	13,618	2,588,862

**Table 17:** Calculation results of monthly fatigue damage of pavement and related parameters.

Month		1	2	3	4	5	6	7	8	9	10	11	12
*T_i_* (°C)		10.4	12.9	18.4	28.1	32.1	34.7	38	34.1	33.4	26	19	12.4
σ (10^−4^ MPa)	Structure A	473	604	753	854	872	879	887	878	876	841	7631	581
	Structure B	665	783	904	974	982	985	986	984	984	966	914	765
	Structure C	755	827	903	938	939	938	936	938	939	935	909	817
k (10^2^)		1013	455	776	3.46	0.95	0.42	0.16	0.50	0.60	6.31	63.1	501
b		3.86	3.94	4.11	4.43	4.56	4.64	4.75	4.62	4.6	4.36	4.13	3.92
*N_f_* (10^9^ times)	Structure A	4310	948	10.7	6.12	2.1	1.1	0.52	1.26	1.44	10.1	86.6	1143
	Structure B	1163	341	51	3.4	1.2	0.65	0.32	0.74	0.85	5.53	41	394
	Structure C	711	274	51	4.1	1.5	0.82	0.41	0.92	1.1	6.4	42	307
*N_i_* (10^6^ times)		5.49	3.7	4.7	3.7	3.79	3.7	3.27	2.16	3.68	4.8	5.77	5.41
*D_i_* (10^−4^)	Structure A	0.006	0.025	0.5	10.5	36.4	74.1	153	37	53.9	7.48	0.72	0.029
	Structure B	0.02	0.07	0.1	18.7	62.6	125	249	6.3	91.4	1.4	1.51	0.082
	Structure C	0.035	0.085	0.1	15.8	50.9	99.4	195	50.7	74	11.9	1.5	0.11

## Data Availability

Data are contained within the article.
